# Assessment of the Efficacy of 0.12% Chlorhexidine Gluconate Mouth Rinse in Preventing Alveolar Osteitis Following Mandibular Molar Extraction

**DOI:** 10.7759/cureus.97889

**Published:** 2025-11-26

**Authors:** Divakar Thiruvenkata Krishnan, Vinej Somaraj, Chethna G, Binsu JM, Azmina Parveen R, Ashni Vibisha AM, Blessy B

**Affiliations:** 1 Oral and Maxillofacial Surgery, Rajas Dental College and Hospital, Tirunelveli, IND; 2 Dentistry, Bharat Institue of Higher Education and Research, Chennai, IND; 3 Public Health Dentistry, Rajas Dental College and Hospital, Tirunelveli, IND

**Keywords:** alveolar osteitis, antiseptic mouthwash, dental prophylaxis, dry socket, early wound healing, re-epithelialization, third molar surgeries

## Abstract

Background: Alveolar osteitis (AO) is a common, painful post-extraction complication, particularly following mandibular molar removal. Clinicians widely recommend 0.12% chlorhexidine (CHX) mouth rinse to prevent AO, but studies on its use in non-surgical extractions with early wound healing assessment are still limited.

Aim: The study aims to assess the efficacy of 0.12% CHX gluconate mouth rinse in preventing AO and enhancing early soft tissue healing after non-surgical mandibular molar extraction.

Materials and methods: A prospective, randomized controlled trial enrolled 60 adult patients, equally allocated to a study group (0.12% CHX rinse, twice daily for seven days starting 24 hours postoperative) and a control group (standard care, no rinse). AO was diagnosed on days three and seven. Early wound healing was evaluated on day seven via the Early Wound Healing Score (EWHS), assessing clinical signs of re-epithelialization (CSR), clinical signs of hemostasis (CSH), and clinical signs of inflammation (CSI). Statistical analysis used Fisher’s exact test for AO incidence and the Mann-Whitney U test for EWHS (p<0.05, significant).

Results: The CHX group recorded zero AO cases (100% success) compared with four cases (13.33%) in controls (p=0.041). Mean EWHS was higher in the study group (7.33 ± 2.12 vs. 6.73 ± 2.36; p=0.198, not significant). About 53.3% of cases in the CHX group achieved excellent healing compared with 36.7% in the control group, with zero poor-healing cases compared with 13.3% in the control group. CSR scores were significantly higher (4.13 vs. 3.60), indicating enhanced epithelialization, while CSH and CSI scores were comparable between groups.

Conclusion: Postoperative use of 0.12% CHX mouth rinse is highly effective in preventing AO and demonstrates a clinically meaningful improvement in early epithelial wound healing. Its simplicity, low cost, and high patient compliance make it an ideal prophylactic strategy for routine clinical implementation.

## Introduction

Alveolar osteitis (AO), or dry socket, is a painful inflammation that usually appears one to three days after a permanent tooth is removed, most often in the lower molars. The condition occurs when the blood clot in the socket breaks down or is lost, leaving bone exposed and causing severe pain with slower healing. Reported incidence varies from about 1% to over 35%, depending on surgical difficulty, operator technique, and patient-related factors. Removing impacted lower third molars carries the greatest risk, with studies showing up to a 10-fold increase compared with routine extractions [1]. Smoking, traumatic surgery, oral contraceptive use, and poor irrigation of the socket further raise the likelihood of this complication [1,2]. Dry socket delays the recovery, leads to extra postoperative visits, and adds to treatment cost, thus prevention remains a priority in oral surgery.

Chlorhexidine (CHX) gluconate is a broad-spectrum antiseptic with strong antibacterial action and long-lasting adherence to oral tissues [1]. By limiting plaque formation and suppressing many oral microbes, it helps reduce the bacterial contamination linked to dry socket. Clinical studies and systematic reviews have tested CHX in various concentrations and delivery forms such as mouth rinses, gels, and bioadhesive preparations for AO prevention. Several studies show that a 0.12% mouth rinse used before or after extraction lowers the incidence of dry socket [3-6]. Meta-analyses also confirm a clear protective effect when CHX is applied as a rinse or placed directly in the socket as a gel [7-11]. Wang et al. [3] reported significant benefit from both 0.12% and 0.2% formulations, and Yengopal et al. calculated a 42%-58 % relative risk reduction with CHX use [7].

Studies on CHX for dry-socket prevention vary widely in concentration, timing, duration, and delivery method [12-14]. Some studies comparing 0.2% gel with 0.12% rinse show higher success with the gel because it stays longer on the tissue [12,15-17]. Other investigations find no meaningful difference between gel and rinse [6,12]. Standardized studies are necessary to test specific formulations and measure patient comfort and early wound healing [18-20]. Data on a fixed 0.12% rinse used only after extraction remain scarce, particularly for its combined effect on dry-socket incidence and early soft-tissue recovery [21,22].

Many of the previous studies have mixed concentrations, tooth types, or have focused only on third molars, which limits how the results apply to routine extractions [5,23,24]. The effect of a single, simple 0.12% CHX rinse after any mandibular molar extraction has not been clearly defined. This aspect remains a serious concern because mandibular molar removal is common worldwide, and even a small drop in dry-socket cases can ease pain, shorten recovery, and reduce follow-up visits. A low-cost rinse that patients can use at home would benefit both high-resource and low-resource settings. This study tests a set protocol of 0.12% CHX mouth rinse twice daily for one week and measures both dry-socket rates and early healing outcomes.

## Materials and methods

Study design and ethical considerations

This prospective, randomized, controlled clinical trial was conducted in the Department of Oral and Maxillofacial Surgery at Rajas Dental College and Hospital. Ethical approval was obtained from the Institutional Ethical Committee (IEC No.: RDCH/PRL/IRB/D-1701/2025), and written informed consent was secured from all participants.

Study population and sample size

A total of 60 adult patients requiring extraction of mandibular molars were recruited and equally allocated into two groups of 30 (N1 and N2: 30 in each). Inclusion criteria were age 18 years or older and a need for the extraction of one or more mandibular molars under local anesthesia. Exclusion criteria included allergy to CHX, bone pathology at the surgical site, smoking, trans-alveolar (surgical) extractions, medical compromise (e.g., uncontrolled diabetes or cardiovascular conditions), bleeding disorders, concurrent antimicrobial therapy, bisphosphonate use, recent radiation therapy to the jaws (within six months), and inability to attend follow-up visits.

Randomization and group allocation

Participants were randomly assigned to one of two parallel groups using a simple randomization method. Group 1 (study group: n=30) received 0.12% CHX gluconate mouth rinse, while group 2 (control group: n=30) received standard postoperative care without antiseptic rinses. Both patients and outcome assessors were blinded to group assignments wherever feasible.

Intervention protocol

Patients in the study group were instructed to rinse with 15 mL of 0.12% CHX gluconate solution twice daily, starting 24 hours after extraction. Each rinse was held in the mouth for 30 seconds and then expectorated. The regimen continued for seven consecutive days. Patients were advised not to eat, drink, or rinse for at least 30 minutes afterward to maximize mucosal contact and efficacy. The control group followed standard postoperative instructions, including analgesics as needed and oral hygiene guidance, without any antiseptic mouthwash.

Clinical procedure and postoperative assessment

All extractions were performed under aseptic conditions by trained clinicians following standardized surgical protocols to minimize trauma. Patients were evaluated on the third and seventh postoperative days. The primary outcome was the incidence of AO, diagnosed by clinical and subjective criteria adapted from Hermesch et al. [1]. Clinical signs included exposed alveolar bone, partial or complete loss of the blood clot, and necrosis of the clot tissue. Subjective symptoms included persistent or increasing throbbing pain beyond 48 hours after extraction. Early wound healing, a secondary outcome, was assessed on day seven using the Early Wound Healing Score (EWHS) of Marini et al. [22], which rates re-epithelialization, hemostasis, and inflammation on a 0-10 scale where 8-10 indicates excellent, 5-7 moderate, and 0-4 poor healing (Table 1).

**Table 1:** Early Wound Healing Score parameters

Parameter	Score	Description
Clinical signs of re-epithelialization	6	Merged incision margins
	3	Incision margins in contact
	0	Visible distance between incision margins
Clinical signs of hemostasis	2	Absence of fibrin on incision margins
	1	Presence of fibrin on incision margins
	0	Bleeding at incision margins
Clinical signs of inflammation	2	Absence of redness along the incision length
	1	Redness involving <50% of the incision length
	0	Redness involving >50% of incision length and/or pronounced swelling

Statistical analysis

Data were analyzed using IBM SPSS Statistics version 22 (IBM Corp., Armonk, NY). Intergroup comparisons of categorical outcomes were performed using the Chi-square test or Fisher’s exact test. Continuous outcomes, such as wound healing scores, were compared using the independent t-test or Mann-Whitney U test based on data distribution. A p-value less than 0.05 was considered statistically significant.

## Results

**Figure 1:**
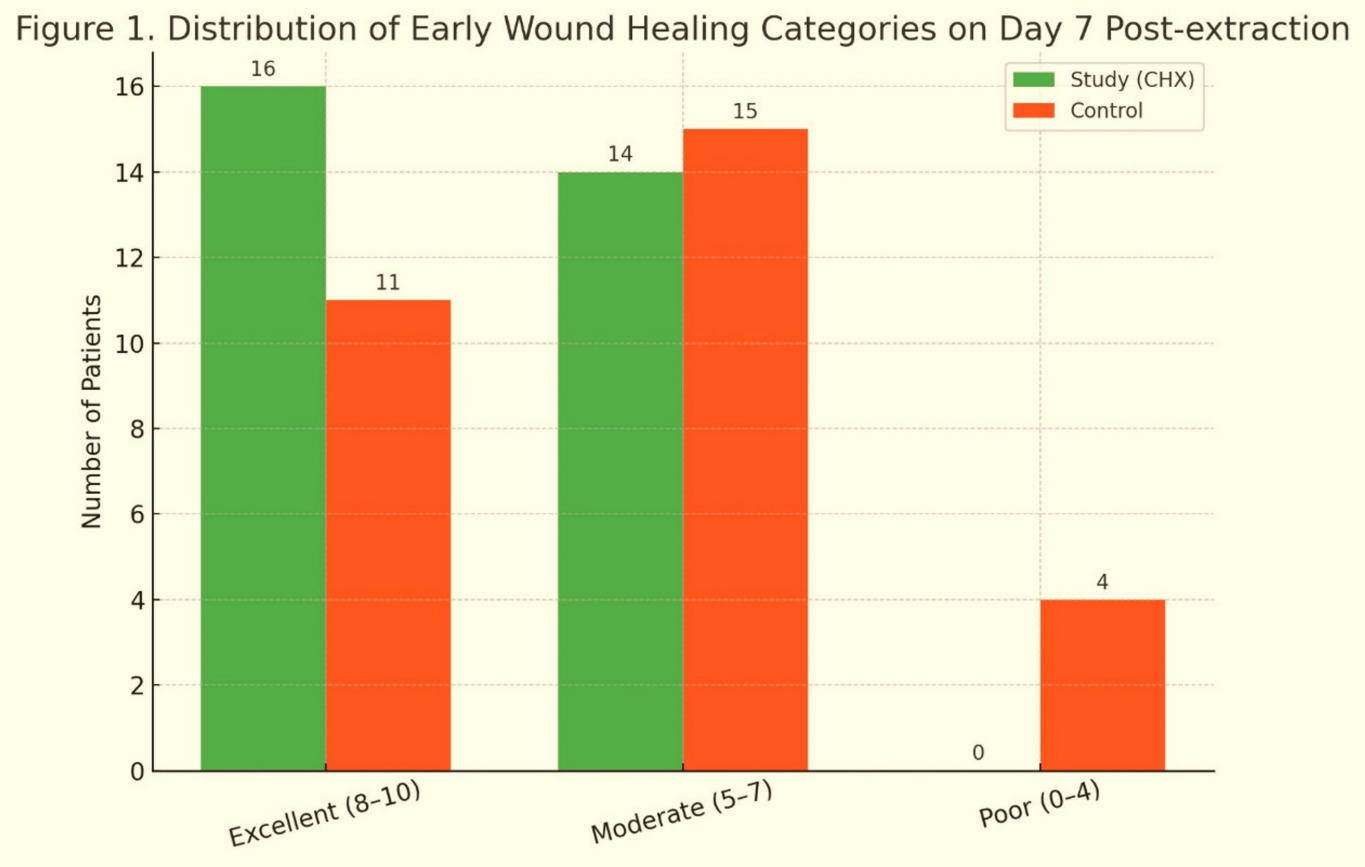
Distribution of early wound healing categories on day 7 post-extraction

Table 2 summarizes the incidence of AO in the study and control groups. In the study group, all 30 patients who used the 0.12% CHX gluconate mouth rinse postoperatively showed no signs of AO, resulting in a 100% success rate. Clinical assessment on the third and seventh postoperative days revealed intact blood clots, no exposed bone, and absence of necrotic tissue, while patients reported no persistent or increasing pain. In contrast, the control group, which received standard postoperative care without an antiseptic rinse, recorded 4 cases of AO among 30 patients, corresponding to an incidence of 13.33% and a success rate of 86.67%. These findings indicate a clear preventive benefit of the CHX rinse in reducing AO occurrence. Table 3 and Figure 1 present a comparative analysis of early wound healing on the seventh postoperative day using the EWHS. The study group, which used a 0.12% CHX rinse, showed a higher mean total EWHS (7.33 ± 2.12) than the control group (6.73 ± 2.36), with median scores of 7 in both groups. In the CHX group, 16 patients (53.3%) achieved excellent healing (scores: 8-10), and 14 patients (46.7%) had moderate healing, with no cases of poor healing. In contrast, the control group had 11 patients (36.7%) with excellent healing, 15 (50.0%) with moderate healing, and 4 (13.3%) with poor healing. These results indicate that CHX use was associated with a higher proportion of excellent healing and elimination of poor-healing outcomes.

**Table 2:** Incidence of AO in study and control groups AO, alveolar osteitis; CHX, chlorhexidine.

Group	Total patients	AO cases	Patients without AO	Success rate (%)
Study group (0.12% CHX rinse)	30	0	30	100
Control group (no CHX)	30	4	26	86.67

**Table 3:** Comparative analysis of total EWHS: Day 7 CHX, chlorhexidine; EWHS, Early Wound Healing Score; IQR, interquartile range; SD, standard deviation.

Parameter	Study group (CHX)	Control group
Mean total EWHS ± SD	7.33 ± 2.12	6.73 ± 2.36
Median (IQR)	7 (6–10)	7 (5–9)
Minimum score	5	2
Maximum score	10	10
Excellent healing (8–10)	16 patients (53.3%)	11 patients (36.7%)
Moderate healing (5–7)	14 patients (46.7%)	15 patients (50.0%)
Poor healing (0–4)	0 patients (0%)	4 patients (13.3%)

**Figure 2:**
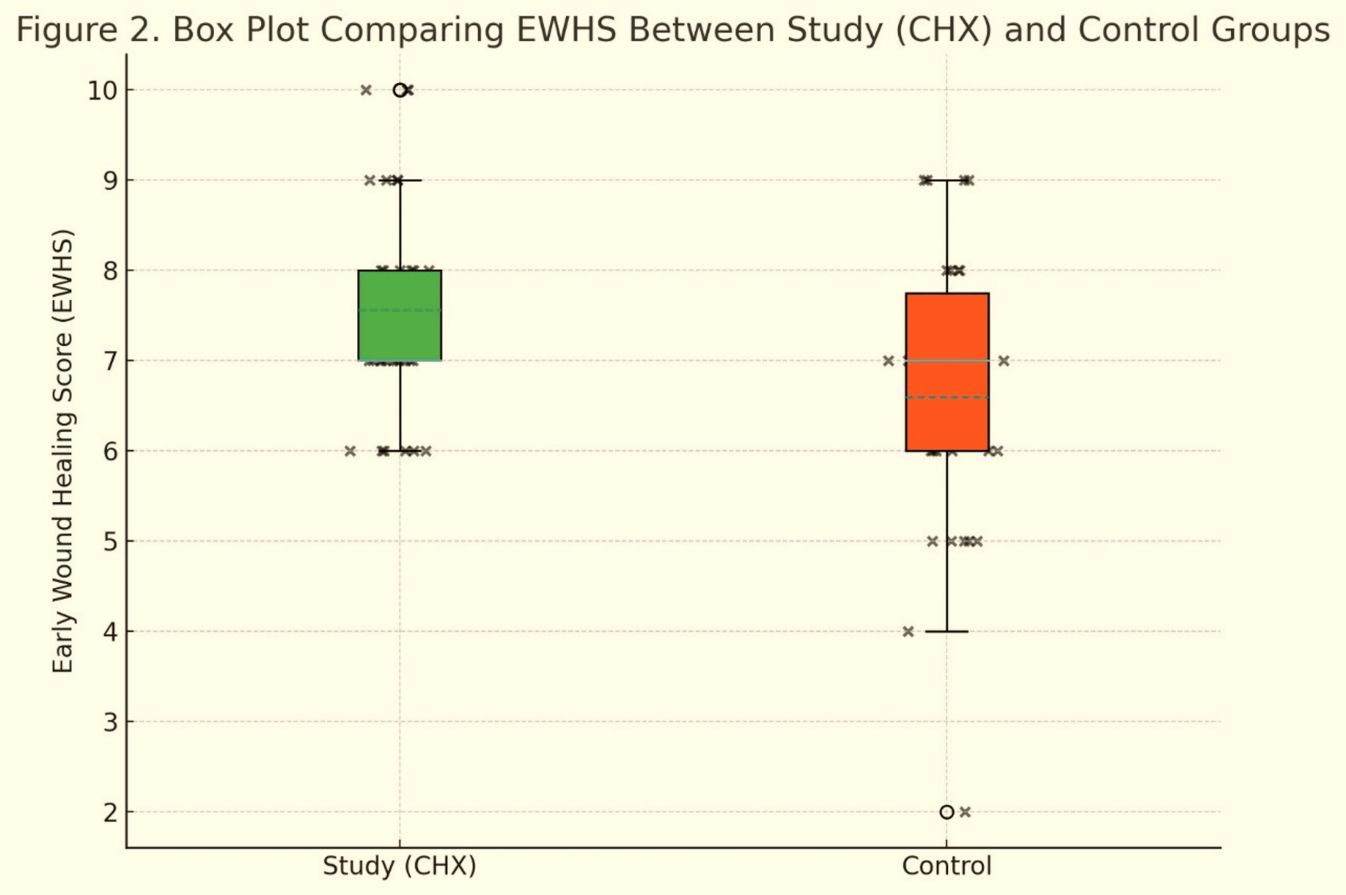
Box plot comparing EWHS between study (CHX) and control groups CHX, chlorhexidine; EWHS, Early Wound Healing Score.

Table 4 and Figure 2 compare the individual EWHS parameters on day 7 between the CHX and control groups. The study group showed a higher mean score for clinical signs of re-epithelialization (CSR: 4.13 ± 1.57) compared with the control group (3.60 ± 1.79), suggesting that a 0.12% CHX rinse may enhance epithelial closure and tissue approximation. Scores for clinical signs of hemostasis (CSH) and clinical signs of inflammation (CSI) were similar between groups (CSH: 1.57 vs. 1.53; CSI: 1.63 vs. 1.60), indicating comparable hemostasis and inflammatory responses. The box plot in Figure 2 visually reinforces these findings, showing a trend toward higher overall EWHS in the CHX group, primarily driven by improved re-epithelialization. Table 5 shows that the study group, using 0.12% CHX rinse, had 0% AO compared with 13.33% in the control group, and Fisher’s Exact Test confirmed this difference as statistically significant (p<0.05), demonstrating the rinse’s preventive efficacy. Table 6 compares EWHS between groups using the independent t-test and Mann-Whitney U test. Although the CHX group had higher mean scores and no poor-healing cases, differences were not statistically significant (p=0.292 and 0.198). Hence, CHX effectively prevented AO, while trends in improved early healing suggest additional clinical benefit.

**Table 4:** EWHS parameter scores (CSR, CSH, and CSI) on day 7 CSH, clinical signs of hemostasis; CSI, clinical signs of inflammation; CSR, clinical signs of re-epithelialization; EWHS, Early Wound Healing Score.

Parameter	Study group	Control group
CSR	4.13 ± 1.57	3.60 ± 1.79
CSH	1.57 ± 0.50	1.53 ± 0.57
CSI	1.63 ± 0.49	1.60 ± 0.50

**Table 5:** Statistical comparison of AO incidence AO, alveolar osteitis.

Comparison	Test used	p-value	Significance
Study vs. control group	Fisher’s exact test	<0.05	Significant

**Table 6:** EWHS statistical comparison EWHS, Early Wound Healing Score.

Test	p-value	Significance	Notes
Independent t-test	0.292	Not significant	Assumes normal distribution
Mann-Whitney U test	0.198	Not significant	Non-parametric; more accurate

## Discussion

The present study demonstrated that postoperative rinsing with 0.12% CHX, started 24 hours after non-surgical mandibular molar extraction and continued twice daily for seven days, prevented every case of AO. None of the 30 participants in the study group developed AO, while the control group showed an incidence of 13.33% (p=0.041). Reported AO rates for similar extractions range from about 1% to more than 30%, depending on population, technique, and diagnostic criteria [3,7,10]. Hermesch et al. found a 19% AO rate in the placebo arm and 6% in the CHX group using comparable non-surgical methods and diagnostic standards [1]. The absolute risk reduction in the present study supports the preventive effect of CHX and is consistent with long-standing clinical evidence that topical use of this antiseptic is a reliable approach for AO prevention.

Randomized comparisons consistently show the benefit of CHX. Wang et al. analyzed 17 trials with more than 2,200 extraction sites and found a pooled risk ratio of 0.43, equal to a 57% relative risk reduction for AO when CHX was used as a rinse or gel [3]. Caso et al. reviewed 14 randomized studies with over 1,600 patients and reported a similar relative risk of 0.42 [10]. Umbrella reviews of these datasets rate the certainty of evidence as moderate to high and show that CHX lowers the risk by about half [11,18,19]. In the present study, no AO cases occurred, a result stronger than the pooled estimates from those analyses. This suggests that a well-controlled rinse protocol can match or even exceed intra-alveolar application when patients follow instructions and the extraction is straightforward.

Studies comparing CHX gel and rinse show similar results. Reported AO rates range from about 3% to 7% with either 0.12% rinse or 0.2% gel, and differences between the two forms have not been statistically significant [12,15,16]. This is supported by a critical appraisal, which concluded that intra-alveolar CHX gel (typically at a 0.2% concentration) is a highly effective intervention for reducing the risk of AO following third molar removal [25]. Reports focused on 0.12% rinse alone usually describe AO incidences between 2% and 8%, with pooled data noting a drop from about 7% in controls to around 3% with rinse use [10]. Using the same diagnostic criteria, one investigation recorded a decrease from 19% in controls to 6% with twice-daily rinsing [1]. The absence of AO in the present non-surgical study fits within or improves on these ranges and does not indicate any advantage for intra-alveolar gel.

EWHS scores showed slightly faster re-epithelialization in the CHX group, but the difference was not statistically significant. In a study of 60 surgical third-molar sites, Amaliya et al. reported a significant increase in soft-tissue closure with 0.2% CHX gel, with mean epithelialization scores about 20% higher than placebo on day seven [21]. A pooled analysis of oral surgery procedures found that CHX provides a small, consistent gain in epithelial migration while having little effect on bleeding or inflammation [11]. In the present study, subscores for bleeding and early inflammation were nearly the same in both groups, supporting the view that CHX mainly promotes epithelial growth and wound edge contact rather than changing clot formation or the acute inflammatory response. Earlier laboratory and clinical reports show that CHX remains active for up to 12 hours, limits bacterial fibrinolysis, and helps preserve the extraction clot needed for rapid epithelial coverage [7,8,10].

Other topical agents show less consistent benefit. Studies of povidone-iodine report AO rates between 8% and 15%, often no better than saline rinsing, and with more local irritation [20]. Hydrogen peroxide rinses give variable results and often cause mucosal discomfort [26]. Intra-alveolar CHX irrigation has been shown to reduce AO more effectively than povidone-iodine after third-molar surgery [27]. A study showed that a novel propolis extract application resulted in an AO rate of 6.25%, which was lower than the 18.75% rate observed in the control group, suggesting it as a promising natural alternative [28]. Warm saline rinses, recommended for mechanical cleansing, provide only limited protection; one study reported AO rates of 12% with saline and 6% with CHX, a difference that was not statistically significant but suggested greater substantivity for CHX [5]. Systematic reviews rank CHX above these alternatives for both efficacy and quality of evidence [18,19].

Studies comparing CHX with systemic antibiotic prophylaxis demonstrate the superiority of topical antiseptics. In a study of 153 third-molar surgeries, Delilbasi et al. compared a 0.2% CHX gluconate rinse to systemic amoxicillin-clavulanic acid and a placebo. They found that CHX was the most effective, with a significantly lower AO rate of 3.9%, compared to 9.8% in the antibiotic group and 13.7% in the placebo group [29]. This supports the use of CHX over routine antibiotics, while also avoiding the risk of antimicrobial resistance. Other reports with different antibiotic regimens reached the same conclusion, noting that local antisepsis with CHX provides equal or better prevention without the adverse effects of systemic drugs [30]. The present study supports these findings by demonstrating complete AO prevention with a simple rinse protocol and no systemic medication. Cost and ease of use add to the rationale for CHX rinsing. A 0.12% CHX rinse costs about ₹54 per patient, less than prescription gels or antibiotic courses. Treating a single AO case often requires several follow-up visits, local curettage, medicated dressings, and analgesics, which create a much higher financial burden for both patients and healthcare services. Twice-daily rinsing is easy to explain, well tolerated, and shows high compliance, as observed in this cohort. With proven efficacy and a strong safety record, 0.12% CHX mouth rinse offers a practical, low-cost option for preventing AO, particularly in settings with limited resources.

Strengths and limitations

This study used strict exclusion criteria, standardized extraction and irrigation techniques, and blinded outcome assessment. These factors improve internal validity and allow clear attribution of the results to the intervention. The main limitation was the modest sample size, which may have limited the power to detect small differences in healing scores. The single-center design and focus on healthy adults undergoing non-surgical extractions restrict generalizability to high-risk or surgically complex cases.

## Conclusions

This randomized clinical trial found that using 0.12% CHX mouth rinse for seven days, beginning one day after non-surgical mandibular molar extraction, prevented all cases of AO and showed a small, non-significant gain in early epithelial healing. The effect was comparable to or greater than that reported in systematic reviews and earlier randomized studies. The rinse was low-cost, simple to use, and well tolerated. These results support prescribing 0.12% CHX rinse as routine postoperative care for standard extractions. Larger multicenter studies that include surgical third-molar cases, smokers, and patients with medical comorbidities are needed to confirm efficacy in higher-risk groups and to evaluate possible benefit from combining the rinse with measures such as socket sealing or cryotherapy.
